# High Throughput, Multiplexed Pathogen Detection Authenticates Plague Waves in Medieval Venice, Italy

**DOI:** 10.1371/journal.pone.0016735

**Published:** 2011-03-10

**Authors:** Thi-Nguyen-Ny Tran, Michel Signoli, Luigi Fozzati, Gérard Aboudharam, Didier Raoult, Michel Drancourt

**Affiliations:** 1 Unité de Recherche sur les Maladies Infectieuses et Tropicales Emergentes (URMITE), UMR CNRS 6236 IRD 198, IFR48, Faculté de Médecine, Université de la Méditerranée, Marseille, France; 2 Anthropologie Bioculturelle, UMR 6578 CNRS, EFS, Université de la Méditerranée, Marseille, France; 3 Soprintendenza Archeologica del Veneto, Venice, Italy; University of Iowa, United States of America

## Abstract

**Background:**

Historical records suggest that multiple burial sites from the 14th–16^th^ centuries in Venice, Italy, were used during the Black Death and subsequent plague epidemics.

**Methodology/Principal Findings:**

High throughput, multiplexed real-time PCR detected DNA of seven highly transmissible pathogens in 173 dental pulp specimens collected from 46 graves. *Bartonella quintana* DNA was identified in five (2.9%) samples, including three from the 16th century and two from the 15th century, and *Yersinia pestis* DNA was detected in three (1.7%) samples, including two from the 14th century and one from the 16th century. Partial *glp*D gene sequencing indicated that the detected *Y. pestis* was the Orientalis biotype.

**Conclusions:**

These data document for the first time successive plague epidemics in the medieval European city where quarantine was first instituted in the 14th century.

## Introduction

The history of Venice, Italy is tightly linked to the ancient plague and particularly to the Second Pandemic, which originated in Europe with the Black Death in the mid-14th century. The commercial activity of the Venetian Republic facilitated trade and interactions with the Southern and Oriental regions of the Mediterranean Sea, where the plague was endemic. Starting in 1348, Venice suffered several plague epidemics, most notably the Black Death [1]. Historical records indicate that a massive epidemic swept through the city during the 14th century [2], which is thought to have killed thousands of people and profoundly affected the history of this prosperous city. Following the initial wave, additional and more detrimental epidemics occurred in 1462, 1485, 1506, 1575–1577 and 1630–1632. In Venice, the number of deaths was first recorded during the 1575–1577 epidemic, with a mortality rate of 27.8%; the 1630–1632 epidemic had a mortality rate of 32.5% of the Venetian population [1], [3].

The cause of these disasters is a matter of debate, and it has not been universally agreed upon that these epidemics were due to *Yersinia pestis* [4]. Alternative hypotheses including influenza [5], anthrax [6] and hemorrhagic fever [7] have been proposed. Using suicide PCR and a recently developed multiplex molecular approach to identify pathogens in ancient human remains [8], we demonstrate here that the Venetian epidemics were indeed plague outbreaks caused by the bacterial species *Y. pestis*.

## Methods

### Archaeological sites

**Figure 1:**
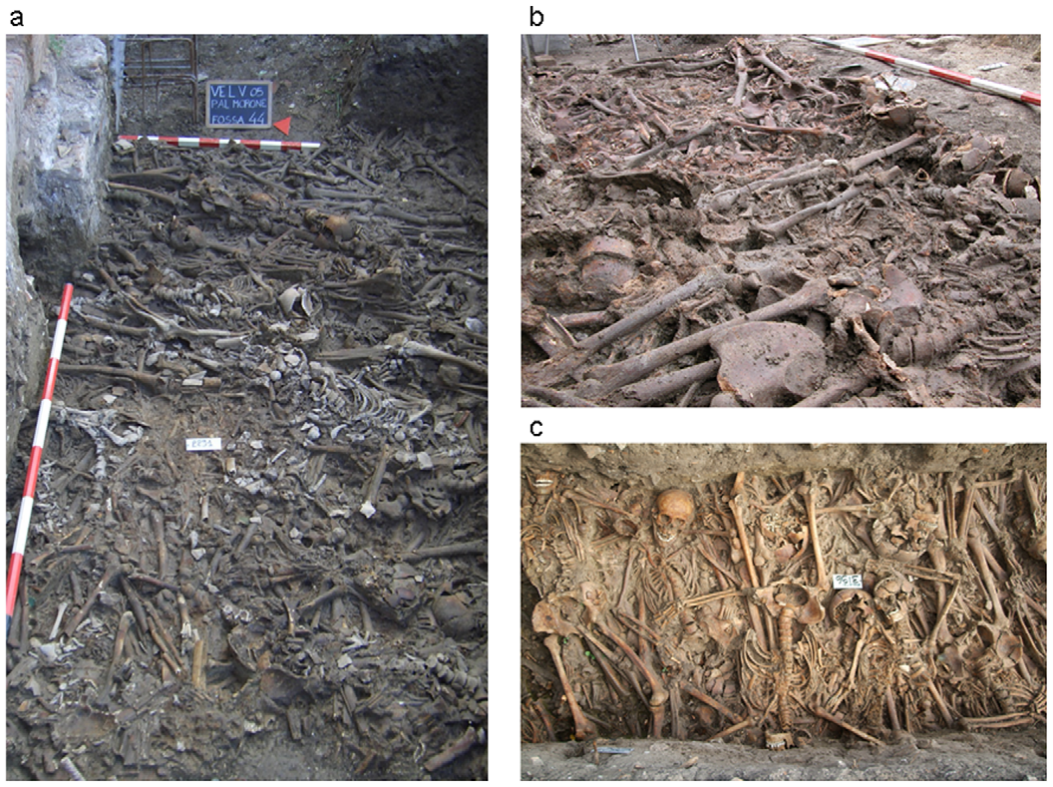
Three views of the medieval plague burial sites in Venice, Italy. a: grave 2; b: grave 35; c: grave 44.

During 2004 and 2005, the renovation of the buildings of Lazzaretto Vecchio in Venice revealed several burial sites containing victims of the plague epidemics (Figure 1). Skeletons from this site were collected by Michel Signoli and Luigi Fozzati. A total of 92 burial locations including graves and trenches were discovered at this site, each containing 5–184 individuals. Pottery fragments found in the sediment were used to determine the age of each site [2], [9]. Sites 21, 24, 26, 34, 90, 91 and 92 dated to the second half of the 14th century and were organized in regular, narrow, parallel graves approximately 50 cm apart. The graves had an east–west or a west–east orientation and were mainly located in the western part of the Prato al Morti. The corpses were deposited in a supine position on the same level. In sites 26 and 34, the bodies were deposited on ceramic (graffita arcaica) dating to the mid-14th century. Burial sites dating to the 15th century could be divided into two major groups. The first consisted of regular, parallel trenches that intersected and often partially or totally destroyed earlier trenches. This suggested that the locations of the previous burial sites were not recorded. The second group consisted of several levels of large graves. Burials dating to the 16th century were in equally large and long trenches. The burials from the early 17th century epidemic were more dispersed and characterized by regular trenches in an east–west orientation or by rectangular graves with varying numbers of corpses.

### Prevention of contamination

Ancient teeth were collected separately from different skeletons in burial sites by archaeologists and transported to the laboratory in individual bags. The dental pulp, which is protected from external contamination in the central cavity and the root canal of the tooth, was used for molecular experiments [10]. The teeth used in this study had closed apexes and were free of caries and trauma. All instruments used to collect dental pulp were sterilized for each tooth to prevent cross contamination, and all reagents were from new kits. The laboratory followed general procedures for decontamination including the use of decontamination solutions and sterilization by ultraviolet light before experiments. PCR experiments were performed according to the suicide PCR protocol previously used for *glp*D by our research team [11]. The experiments were done in a laboratory where *Y. pestis* and *Y. pestis* DNA have not been previously handled. Ancient teeth collected from corpses devoid of any anthropological evidence of infection were collected from a cemetery in Moirans, France (16th–18th) in agreement with French regulations and with appropriate permission of French authorities; they were used as negative controls in the PCR analyses.

### High throughput detection of pathogens

Dental pulp was recovered as previously described [12] and incubated overnight at 56°C with 600 µL of ATL buffer and 50 µL of proteinase K. The total DNA was extracted using the QIAamp Media MDx Kit and pulverized on the BioRobot® MDx workstation in a final volume of 100 µL (Qiagen GmbH, Hilden, Germany). The high throughput detection of seven pathogens was performed as previously described [13]. Briefly, DNA of *Y. pestis*, *Bacillus anthracis* (anthrax agent), *Borrelia recurrentis* (louse-borne relapsing fever agent), *Bartonella quintana* (trench fever agent), *Rickettsia prowazekii* (epidemic typhus agent), *Salmonella enterica* Typhi (typhoid fever agent) and poxvirus (smallpox agent) (Table) was detected with high throughput multiplexed real-time PCR. Two wells containing sterile water and two containing DNA extracted from dental pulp collected from negative control corpses served as standards.

**Table 1:** Primers and probes for the molecular detection of pathogens in ancient teeth.

Pathogen	Gene	Probe and primers	PCR product lenght
*Bacillus anthracis*	*pag*	6 FAM- TAC CGC AAA TTC AAG AAA CAA CTG C -TAMRA	94 bp
		5′- AGG CTC GAA CTG GAG TGA A -3′	
		5′- CCG CCT TTC TAC CAG ATT T -3′	
*Borrelia recurrentis*		6 FAM- CTG CTG CTC CTT TAA CCA CAG GAG CA -TAMRA	111 bp
		5′- TCA ACT GTT TTT CTT ATT GCC ACA -3′	
		5′- TCC TTA TGT TGG TTA TGG GAT TGA -3′	
*Bartonella quintana*	ITS	6 FAM- GCG CGC GCT TGA TAA GCG TG -TAMRA	102 bp
		5′- GAT GCC GGG GAA GGT TTT C -3′	
		5′- GCC TGG GAG GAC TTG AAC CT -3′	
*Rickettsia prowazekii*	*ompB*	6 FAM- CGG TGG TGT TAA TGC TGC GTT ACA ACA -TAMRA	134 bp
		5′- AAT GCT CTT GCA GCT GGT TCT -3′	
		5′- TCG AGT GCT AAT ATT TTT GAA GCA -3′	
*Salmonella enterica* Typhi		6 FAM- GCT TTT TGT GAA GCA ACG CTG GCA -TAMRA	138 bp
		5′- CTC CAT GCT GCG ACC TCA AA -3′	
		5′- TTC ATC CTG GTC CGG TGT CT -3′	
Poxvirus	HA	6 FAM- AAG ATC ATA CAG TCA CAG ACA CTG T -TAMRA	100 bp
		5′- GAC KTC SGG ACC AAT TAC TA -3′	
		5′- TTG ATT TAG TAG TGA CAA TTT CA -3′	
*Yersinia pestis*	*pla*	6 FAM- TCC CGA AAG GAG TGC GGG TAA TAG G -TAMRA	98 bp
		5′- ATG GAG CTT ATA CCG GAA AC -3′	
		5′- GCG ATA CTG GCC TGC AAG -3′	

### *Y. pestis* DNA genotyping

Further genotyping of *Y. pestis* was based on suicide PCR of the *glp*D gene [11]. A previously reported *glp*D primer pair [11] was used and the PCR was conducted in a laboratory in which *Y. pestis* and *Y. pestis* DNA were not previously handled. The PCR products were separated by electrophoresis at 100 V in a 2% agarose gel and sequenced using the Big Dye Terminator Kit. Sequencing products were resolved with the ABI PRISM 3130 Genetic Analyzer (Applied BioSystems, Courtaboeuf, France) and analyzed with the ABI PRISM DNA Sequencing Analysis Software version 3.0 (Applied BioSystems). Sequences were compared with those available in the GenBank database by BLAST (http://www.ncbi.nlm.nih.gov/blast/Blast.cgi).

## Results

### High throughput detection of pathogens

A total of 173 dental pulp specimens from Venice were analyzed including 37 specimens dating to the 14th century, 45 from the 15th century, 48 from the 16th century and 43 from the 17th century. Negative controls were negative in all experiments. High throughput real-time PCR detected *B. quintana* DNA in five (2.9%) dental pulp specimens, including three from the 16th century and two from the 15th century, and *Y. pestis* DNA was detected in three (1.7%) specimens, including two from the 14th century and one from the 16th century. The other five tested pathogens were not detected in this study.

### *Y. pestis* DNA genotyping

The presence of *Y. pestis* DNA was confirmed by amplifying 165 bp of the *glp*D gene in two specimens, including one specimen positive by real-time PCR (from grave 35) for *Y. pestis* and another specimen negative by real-time PCR. The sequence of the PCR product derived from the specimen of grave 35 was most closely related to that of the *Y. pestis* biotype Orientalis *glp*D gene (GenBank accession number AL59082) with 98% sequence similarity. This sequence is characterized by a 93-bp deletion compared with the *glp*D gene sequence of *Y. pestis* Antiqua (GenBank accession number NC008150).

## Discussion

The results reported here are authentic; the negative controls remained negative in the two rounds of PCR-based experiments, and *Y. pestis* was specifically detected using two independent PCR-based experiments including suicide PCR. The specificity of the PCR products was further confirmed by sequencing [10].

The innovative approach used in this study was based on high throughput, multiplexed detection of seven pathogens that have been implicated in several epidemics with high mortality rates [14]. Previous studies reported the detection of bacteria in the dental pulp of buried individuals [12], [15]. This multiplexed approach allowed the detection of two organisms in individuals recovered from the same grave. *B. quintana* is a blood-borne organism and the etiological agent of trench fever resulting from bacteremia [16]. However, asymptomatic bacteremia has also been reported [17] indicating that only the detection of *B. quintana* DNA in the dental pulp does not definitively identify the cause of death in ancient, buried individuals. However, the same is not true for *Y. pestis*; untreated septicemia always results in death [18], [19]. Therefore, we interpreted the detection of *Y. pestis* DNA as indicative that these individuals died of septicemic plague. This approach eliminated five pathogens previously implicated without any experimental evidence as being responsible for the Black Death [8]. Only *B. quintana* and *Y. pestis* were detected in these Venetian individuals.

*B. quintana* has previously been detected in human remains including a Neolithic individual [20] and in Napoleon Great Army soldiers from 1815 who also had typhus [21]. We recently detected a *B. quintana* and *Y. pestis* co-infection in individuals excavated from a burial site near Paris dating to the 11th–15th centuries (Drancourt and Le Forestier, unpublished data). *B. quintana* is transmitted by the human body louse *Pediculus humanus* [22], which has been experimentally demonstrated to carry *Y. pestis* [23], [24] and was observed during familial plague outbreaks [25]–[27]. Medieval populations are known to have been largely infested by body lice and the observation here of a co-infection with *B. quintana* and *Y. pestis* is compatible with the hypothesis that the body louse was a vector driving the Black Death epidemics in Europe [28], [29].

**Figure 2:**
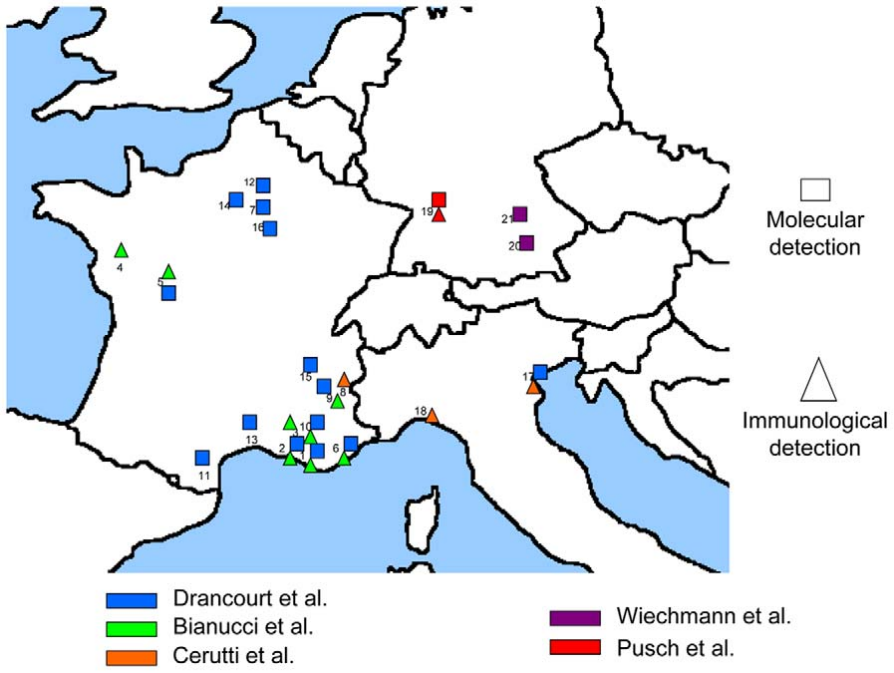
Molecular (squares) and immunological (triangles) detection of the plague agent *Yersinia pestis* in ancient burial sites in Europe made by six teams (Marseille team, blue). References are indicated in brackets. **France:** 1. Marseille (18th) [11], [12], [36]; 2. Martigues (18th) [11], [36]; 3. Berre l'Etang (18th) [37]; 4. La Chaize-le-Vicomte (17th–18th) [38]; 5. Poitiers (16th–18th) [38] (Drancourt, unpublished data); 6. Draguignan (17th) [36] (Drancourt, unpublished data); 7. Saint-Maurice (17th) [41]; 8. Briançon (17th) [35]; 9. Lariey (17th) [37] (Drancourt, unpublished data); 10. Lambesc (16th) [12], [36]; 11. Vilarnau (13th–15th) [30]; 12. Bondy (11th–15th) [Drancourt, unpublished data]; 13. Montpellier (13th–14th) [8], [31]; 14. Dreux (12th–14th) [31]; 15. Vienne (7th–9th) [11]; 16. Sens (5th–6th) [31]; 17. Saint-Laurent-de-la-Cabrerisse (AD 1348 or 1374) [32]. **Italy:** 18. Venice (14th–17th) [present study]; 19. Genoa (Bastione dell'Acquasola) (14th) [35]; 20. Parma (16th/17th) [32]. **Germany:** 21. Stuttgart (14th–17th) [33]; 22. Aschheim (6th) [42]; 23. Manching-Pichl (Late medieval) [34]; 24. Augsburg (16th/17th) [32]; **The Netherlands:** 25. Bergen op Zoom (Mid-14th) [32]. **England:** 26. Hereford (AD 1335±54) [32].

Our results detail the start of the Black Death in Europe in the mid-14th century. Several works previously documented *Y. pestis* in human remains from the Black Death (Figure 2) including *Y. pestis* DNA in one individual in Vilarnau, France from the 13th–15th centuries [30], one individual from the second half of the 14th century in the Saint Come and Saint Damien sites in Montpellier, France [8], three individuals in Dreux, France from the 12th–14th centuries [31], one individual in Saint-Laurent-de-la-Cabreisse, France from the AD 1348 or 1374 [32], two individuals in Bondy, France from the 11th–15th centuries (Drancourt and Le Forestier, unpublished data), two individuals in Stuttgart, Germany from the 14th–17th centuries [33], five late medieval individuals in Manching-Pichl, Germany [34], seven individuals in Bergen op Zoom, the Netherlands from the mid-14th century (AD 1349-50) and two individuals in Hereford, England from the AD 1335±54 [32]. In addition, immunological detection of the F1 antigen has been reported in seven individuals in Saint-Laurent-de-la-Cabreisse, France [32], one individual in Genoa, Italy from the 14th century [35], ten individuals of Stuttgart, Germany from the 14th–17th centuries [33], three individuals in Bergen op Zoom, the Netherlands and four individuals in Hereford, England [32]. *Y. pestis* has been documented in ten Black Death burial sites scattered over five countries by using different methodological approaches, and therefore the Black Death undoubtedly was due to the plague agent *Y. pestis* [32]. In the present study, ancient *Y. pestis* DNA has been detected in only a small proportion of buried individuals in agreement with previous studies, indicating that detection of aDNA lacked sensitivity, in contrast to the immunological detection of the *Y. pestis* F1 antigen [32], [33], [36]–[38]. One Black Death site yielded 10/12 (83.3%) positives in the F1 dipstick assay and only 2/12 (16.7%) positives with PCR techniques [33]. Another recent study yielded only 10/72 (14%) positives with PCR and 24/47 (51%) positives by the F1 dipstick assay [32]. Molecular techniques allowed for genotyping ancient plague and yielded *Y. pestis* Orientalis on the basis of multiple spacer sequencing [31] and a characteristic deletion in the *glp*D gene as in Venice [11]. A recent analysis of single nucleotide polymorphisms yielded two previously unknown, non-Orientalis clades of *Y. pestis* in South France, in the Netherlands and in England [32]. In latter study, plague in 17th century Parma, another North Italy city was ascertained by immunological detection of the F1 antigen but aDNA detection failed and genotyping was not done.

The originality in the organization of the Lazzaretto Vecchio site is owed to the fact that, unlike other plague burial sites investigated to date; this location was utilized during the Venetian plague waves for four centuries rather than only a single epidemic. This site contains multiple, simultaneous burial sites from different periods of major demographic crises that reflect the unique management of an epidemic. In Venice, the island of Santa Maria di Nazareth appears to have been used since the beginning of the Second Pandemic, if not for the care, at least for the burial of victims.

While the Black Death significantly affected Venice, this medieval city imposed the most efficient prevention measures of the time by increasing the 30-day isolation decreed in Ragusa (currently Dubrovnik) to a 40-day isolation known as quarantine [39]. Shortly, all of the port cities in medieval Europe set up quarantine areas that persisted until the 20th century [40].
